# TMSB4 Overexpression Enhances the Potency of Marrow Mesenchymal Stromal Cells for Myocardial Repair

**DOI:** 10.3389/fcell.2021.670913

**Published:** 2021-06-09

**Authors:** Shiyuan Tang, Chengming Fan, Chukwuemeka Daniel Iroegbu, Wenwu Zhou, Zhigong Zhang, Ming Wu, Wangping Chen, Xiaoming Wu, Jun Peng, Zhihong Li, Jinfu Yang

**Affiliations:** ^1^Department of the Cardiovascular Surgery, The Second Xiangya Hospital, Central South University, Changsha, China; ^2^Department of the Cardiovascular Surgery of the Hunan Provincial People’s Hospital, Changsha, China; ^3^Hunan Provincial Key Laboratory of Cardiovascular Research, Changsha, China; ^4^Institute of Senile and Aging Diseases, The Second Xiangya Hospital of Central South University, Changsha, China

**Keywords:** thymosin beta-4, hypoxia-inducible factor-1α, mesenchymal stromal cell, angiogenesis, heart failure, AKT, YC-1

## Abstract

**Objective:**

The actin-sequestering proteins, thymosin beta-4 (Tβ4) and hypoxia-inducible factor (HIF)-1α, are known to be associated with angiogenesis after myocardial infarction (MI). Herein, we aimed to identify the mechanism of HIF-1α induction by Tβ4 and investigate the effects of bone marrow mesenchymal stromal cells (BMMSCs) transfected with the Tβ4 gene (*TMSB4*) in a rat model of MI.

**Methods:**

Rat BMMSCs were isolated, cultured, and transfected with the *TMSB4* gene by using the lentivirus-mediated method. Rats with surgically induced MI were randomly divided into three groups (*n* = 9/group); after 1 week, the rats were injected at the heart infarcted border zone with TMSB4-overexpressed BMMSCs (BMMSC-TMSB4^*OE*^), wild-type BMMSCs that expressed normal levels of TMSB4 (BMMSC-TMSB4^*WT*^), or medium (MI). The fourth group of animals (*n* = 9) underwent all surgical procedures necessary for MI induction except for the ligation step (Sham). Four weeks after the injection, heart function was measured using transthoracic echocardiography. Infarct size was calculated by TTC staining, and collagen volume was measured by Masson staining. Angiogenesis in the infarcted heart area was evaluated by CD31 immunofluorescence histochemistry. *In vitro* experiments were carried out to observe the effect of exogenous Tβ4 on HIF-1α and explore the various possible mechanism(s).

**Results:**

*In vivo* experiments showed that vascular density 4 weeks after treatment was about twofold higher in BMMSC-TMSB4^*OE*^-treated animals than in BMMSC-TMSB4^*WT*^-treated animals (*p* < 0.05). The cardiac function and infarct size significantly improved in both cell-treatment groups compared to controls. Notably, the cardiac function and infarct size were most prominent in BMMSC-TMSB4^*OE*^-treated animals (both *p* < 0.05). HIF-1α and phosphorylated HIF-1α (p-HIF-1α) *in vitro* were significantly enhanced by exogenous Tβ4, which was nonetheless blocked by the factor-inhibiting HIF (FIH) promoter (YC-1). The expression of prolyl hydroxylase domain proteins (PHD) was decreased upon treatment with Tβ4 and further decreased with the combined treatment of Tβ4 and FG-4497 (a specific PHD inhibitor).

**Conclusion:**

TMSB4-transfected BMMSCs might significantly improve recovery from myocardial ischemia and promote the generation of HIF-1α and p-HIF-1α *via* the AKT pathway, and inhibit the degradation of HIF-1α *via* the PHD and FIH pathways.

## Introduction

The mortality rate of myocardial infarction (MI) is positively associated with the infarct size. As the functionality of cardiomyocytes proliferation is limited in an adult mammal heart, promotion of angiogenesis remains the most crucial strategy in salvaging myocytes at the infarcted border zone. Thus, discovering practical or appropriate clinical interventions, sparing less severely damaged myocytes at border zones, could effectively reduce infarct size and save lives (Fan et al., 2020a). Mesenchymal stromal cells (MSCs) represent a promising tool for cell therapy, particularly for heart-related diseases. The essential mechanisms include preserving myocardial contractility, modulating fibrosis, and promoting angiogenesis (White and Chong, 2020).

Cell-based therapies for MI using MSC-derived exosomes are well studied owing to their strong pro-angiogenic effect. Genetic modification is one of the most common methods used to enhance exosome therapy (Sun et al., 2020), and vascular endothelial growth factor (VEGF), fibroblast growth factor (FGF), hypoxia-inducible factor (HIF)-1α, and thymosin beta-4 (Tβ4) have been identified as the most promising candidates (Fan et al., 2020a,b; Sun et al., 2020; Ye et al., 2013). The transcription factor HIF-1 plays an important role in cellular response to systemic oxygen levels in mammals, and its activity is age dependent. Mouse studies suggest that aging impairs ischemia-induced vascular remodeling by inhibiting the induction of HIF-1 and its downstream target genes, thereby blocking both the production of angiogenic signals and the ability of bone marrow–derived angiogenic cells (BMDACs) to respond to them (Rey et al., 2009). Combined HIF-1α-based gene and cell therapy reduced tissue necrosis even when BMDAC donors and ischemic recipient mice were 17 months old, suggesting that this approach may have therapeutic utility in elderly patients with critical limb ischemia. Tβ4 is known to be involved in angiogenesis as a pro-angiogenic and fibroblast-activating peptide (Qian et al., 2012). Significantly, Tβ4 was identified as essential for all aspects of coronary vessel development in mice (Smart et al., 2007a).

It is believed that Tβ4 induces angiogenesis by increasing the expression of growth factors such as HIF-1α and stabilizing HIF-1α protein levels in an oxygen-independent manner (Jo et al., 2010; Ock et al., 2012). However, the mechanism of HIF-1α expression and Tβ4-induced degradation largely remains unknown. Herein, we aimed to identify the mechanism of HIF-1α induction by Tβ4 and investigate the effects of bone marrow mesenchymal stromal cells (BMMSCs) transfected with the Tβ4 gene (*TMSB4*) in a rat model of MI.

## Materials and Methods

### Isolation and Cultivation of MSCs From Bone Marrow of Sprague–Dawley Rats

Sprague–Dawley rats were purchased from the Department of Experimental Animal Center, Second Xiangya Hospital, Central South University, Changsha, China. All animals received humane care in compliance with the “Guide for the Care and Use of Laboratory Animals” prepared by the Institute of Laboratory Animal Resources, National Research Council, and published by the “Guide to the Care and Use of Experimental Animals” by the Chinese Council on Animal Care. BMMSCs were isolated in a lymphocyte separation medium and by density gradient centrifugation as previously described (Yang et al., 2009; Tang et al., 2013).

Four-week-old Sprague–Dawley female rats (weight, ∼100 g) were selected. The cells were isolated from the bone marrow of upper and lower limb bones and separated by gradient centrifugation with 1.073 g/ml Percoll solution (Promega, United States). The cells were cultured in Dulbecco’s Modified Eagle’s medium (DMEM, Gibco, United States) containing 15% fetal bovine serum, 1 ng/ml basic fibroblast growth factor, and 200 mmol/L glutamine at 37°C in a humidified atmosphere containing 5% carbon dioxide (Forma, United States).

Renew half of the culture medium for the first 8 h and then replace whole medium with fresh DMEM every 3 days. Purified BMMSCs were observed after four times of medium exchange. For morphological observations, the cells were inoculated in a 60-mm culture dish at a density of 1 × 10^7^/cm^2^. The first, third, fifth, and seventh passage of cells were selected and counted. The cell numbers from days 1 to 14 and each passage’s growth curves were generated and analyzed. Cells from passages 3 to 8 were used for the study.

### TMSB4 Transfection Into MSCs

Lentivirus plasmids and TMSB4-pLent-GFP-Puro-CMV were purchased from ViGene Biosciences (Shandong, China). Extraction and identification of plasmids were performed according to the manufacturer’s recommendations. In brief, TMSB4 (NM_031136: ATGTCTGACAAACCCGATATGGCTGAGATCGAGAAATTC GATAAGTCGAAGTTGAAGAAGACAGAAACACAAGAGAA AAATCCTCTGCCTTCAAAAGAAACAATTGAACAAGAGA AGCAAGCTGGCGAATCGTAA) was amplified by polymerase chain reaction (PCR) and then recombined into the target vector—pLent-GFP-Puro-CMV (Asis1-Mlu1 enzyme digestion vector)—to obtain the full-length construction of *TMSB4* gene. First, 24 h before transfection, the fifth generation of MSCs (∼70–80% confluent) was digested by 0.05% Trypsin and 0.02% EDTA. Second, the MSCs were then vaccinated onto 12-pore plates using an opioid sterilized round cover glass (∼1 × 10^5^ MSCs/pore, each pore containing 1 ml L-DMEM culture solution with 15% fetal bovine serum). Finally, the MSCs were cultured in the traditional incubator with 5% carbon dioxide at 37°C in a saturated humidified atmosphere.

The MSCs were transfected with lentiviral supernatants of 0, 1, 2.5, and 5 μl. The culture medium was completely replaced after 24 h. The expression of fluorescent protein in the transfected cells was observed under a fluorescence microscope after 72 h.

The transfection efficiency of BMMSCs was observed under a confocal microscope after 7 days of co-culture. Puromycin (5 μg/ml) was used for the selection and maintenance of cell lines.

The TMSB4-overexpressing BMMSCs (BMMSC-TMSB4^*OE*^) were used for flow cytometric (FACSort, B-D Co., United States) analysis to detect the cellular markers including CD90, CD29, CD45, CD34, CD11B, CD105, CD73, HLA-DR, and CD19. MSCs were gathered and diluted using PBS at a concentration of 10^6^ cells/ml. After incubating with fluorescence-labeled antibodies for 15 min at room temperature, cells were then washed twice with PBS and dispersed to make a single-cell suspension. The tripotent differentiation, including osteogenesis, chondrogenesis, and adipogenesis, were induced according to previously described methods (Yang et al., 2007, 2009; Tang et al., 2013).

### Detection of *TMSB4* Expression in the Target Cells

*GFP*, a marker gene, would be expressed automatically along with the target gene. Thus, the expression of fluorescent-labeled *GFP* was considered representative for the expression of *TMSB4*. The transfected cells were observed and detected at different time points. The expression of *GFP* was observed using an inverted microscope with an excitation wavelength of 490 nm. Western blot assay was used to explore the expression of *T*β*4*, *HIF-1*α, *p-HIF-1*α, *p-AKT*, and *VEGF* in transfected MSCs.

### Experimental Animals

Surgical induction of MI was performed on female Sprague–Dawley rats. In brief, rats were intubated and breathing *via* a ventilator with 2% isoflurane USP (Fluriso^TM^, VetOne) to maintain anesthesia^TM^. After a thoracotomy was performed *via* the left fourth intercostal space, the anterior descending branch of the left coronary artery (LAD) was surgically ligated using a 6–0 suture. Thirty animals were used to establish the MI model. However, 3/30 rats died due to peri-/post-operative complications.

The surviving animals were randomly divided into three groups (*n* = 9/group). After the first week of MI, the rats were injected with the fifth passage of TMSB4-overexpressing BMMSCs (BMMSC-TMSB4^*OE*^), the same passage of wild-type BMMSCs that expressed normal levels of TMSB4 (BMMSC-TMSB4^*WT*^), or the same volume (150 μl) of medium (MI) at three different sites (1 × 10^6^ cells/50 μl/site) in the border zone of the anterior wall of the left ventricle (LV) (3 × 10^6^ cells/150 μl/animal). The fourth group of animals (*n* = 9) underwent all surgical procedures necessary for MI induction except for the ligation step (Sham).

### Western Blot Assay

Protein concentration was detected by the BCA protein assay kit (Solarbio life sciences, Beijing, China) according to the manufacturer’s protocols. A 2 × sample buffer was added to an equivalent sample according to the protein concentration denatured at 100°C for 5 min. SDS-PAGE electrophoresis (25 μg/pore) was then performed at a constant voltage of 120 V. After that, the protein was transferred to polyvinylidene difluoride (PVDF) membranes (Trans-Blot^®^ Turbo^TM^ Mini PVDF Transfer Packs, Bio-Rad) at 120 V for about 2 h. Subsequently, the membrane was blocked by a blocking buffer [5% dried skim milk, 25-mm Tris-buffer saline (TBS)] for 2 h, followed by incubation with primary antibodies at 4°C overnight.

Next, the membrane was washed thrice with TBST (for approximately 10–15 min each time), incubated with horseradish peroxidase [(HRP)-conjugated secondary antibody (diluted 1:1,000)] at room temperature for 1 h, and washed again. Finally, an ECL reagent was added, and the membrane was exposed. Western blot signals were measured by densitometry and analyzed using software (AlphaView SA software 3.4, ProteinSimple). The housekeeping protein β-actin was used for Western blot normalization.

### Immunostaining and Fluorescence Microscopy

Following the different treatment methods used, the rat hearts were harvested on the 28th day and processed according to previously described methods (Fan et al., 2020a). Briefly, hearts were fixed with 4% paraformaldehyde at 4°C for 4 h, followed by immersion in 30% sucrose at 4°C overnight. Then, 10-μm-thick serial cryosections were obtained, and every 30th section was selected and permeabilized with 0.2% Triton X-100 for 10 min at room temperature. The sections were blocked in 5% donkey serum in DPBS at a pH of 7.4 for 30 min at room temperature before different antibodies were used.

Primary antibodies were diluted 1:100–1:1,000 with the blocking buffer (1.5% BSA, 100 mM glycine in PBS) and incubated at 4°C overnight. Secondary antibodies (Jackson ImmunoResearch Laboratory) were diluted 1:200 with the blocking buffer and incubated in the dark for 2 h at room temperature. Nuclei were stained or co-stained with 4,6-diamidino-2-phenyl-indole (DAPI, 100 ng/ml, Sigma-Aldrich). Negative controls were stained with only secondary antibodies. The stained sections were analyzed using a fluorescence microscope.

### Echocardiography

Heart function from pre- and post-MI rats (1 and 4 weeks after intervention) were detected by transthoracic echocardiography as previously described (Tang et al., 2013; Fan et al., 2020a). In short, rats were maintained under 1.5–2% isoflurane USP (Fluriso, VetOne) anesthesia until the heart rate was stabilized at 500–700 beats per minute. The two-dimensional M-mode and B-mode images were acquired from both parasternal short- and long-axis views with a high-resolution ultrasound system (Vevo 2100, VisualSonics, Inc.). Finally, the heart beats were recorded and the functional parameters including left ventricular ejection fraction (EF) and fractional shortening (FS) were calculated from several short-axis views using a modified Simpson’s rule and the Vevo analysis software. The operator was blinded to the experimental groups.

### TTC Staining and Determination of Infarct Size

Following the different treatment methods used, on the 28th day, the hearts were excised under deep anesthesia and the infarct sizes were evaluated. Briefly, the freshly harvested heart tissue was cut into five slices using a rodent heart section mold. To maximize saving the heart tissue, each slice was cut down to a thinner slice (1 mm per slice) and used for TTC staining. The remnants were used for IHC and IF (Supplementary Figure 1). Tissues were then placed in 1% TTC solution (Solarbio, Cat: G3005) and incubated at room temperature in the dark for 15 min. The stained tissues were then photographed under a light microscope (Olympus).

Digital images of the stained sections were captured to assess the changes of infarct size at post-treatment day 28. Morphometric analyses were carried out using NIH Image J software. The infarcted size was calculated according to the formula: infarct size (%) = [sum of (scar circumferential length × thickness of each of the short axis)/sum of (short axis left ventricle length × thickness of the short axis)] × 100%.

### Masson and DAB Staining

The explanted hearts were collected on day 28 post-treatment. The tissue was fixed with 4% paraformaldehyde at 4°C for 4 h and then immersed in 30% sucrose at 4°C overnight. Ten-micrometer-thick serial cryosections were obtained, and every 30th section was selected and permeabilized with 0.2% Triton X-100 for 10 min at room temperature. Masson Trichrome Kit and Tunel Cell Apoptosis Detection Kit were purchased from Thermo Fisher Scientific (Cat: 87019) and Servicebio (G1507-20T), respectively. The staining was performed according to the manufacturer’s recommendations. The volume fraction of interstitial collagen was calculated as the ratio of the fibrotic area to the total surface area of the left ventricle. Intramural vessels, perivascular collagen, endocardium, and trabeculae were excluded from this particular analysis. The apoptotic cells were quantified as the number of TUNEL-positive cells divided by the total number of cells and expressed as a percentage (six views per slice and five slices per heart were analyzed).

### Antibodies and Reagents

**Table:** 

Primary antibodies	Cat. No.	Source	Dilutions
Thymosin β4	ab14334	Abcam	1:1,000
HIF	20960-1-AP	Proteintech	1:200
P-HIF	3434S	CST	1:1,000
VEGF	ab1316	Abcam	1:1,000
p-AKT	9275s	CST	1:1,000
PHD	ab108980	Abcam	1:1,000
FIH	4426s	CST	1:1,000
Sarcomeric Alpha Actinin (αSA)	ab9465	Abcam	1:100
CD31	ab182981	Abcam	1:100
CD34-Alexa Fluor^®^ 647	a187283	Abcam	10 μl for 10^6^ cells
Isotype Control	ab176103	Abcam	20 μl for 10^6^ cells
CD11B-PE	201807	Biolegend	0.2 μg for 10^6^ cells
Isotype Control	400211	Biolegend	0.2 μg for 10^6^ cells
CD29-PE	102207	Biolegend	0.2 μg for 10^6^ cells
Isotype Control	400907	Biolegend	0.2 μg for 10^6^ cells
CD45-FITC	202205	Biolegend	0.2 μg for 10^6^ cells
Isotype Control	400107	Biolegend	0.2 μg for 10^6^ cells
CD90-PE	205903	Biolegend	0.2 μg for 10^6^ cells
Isotype Control	400311	Biolegend	0.2 μg for 10^6^ cells
β-actin	60008-1-Ig	Proteintech	1:5,000

**Reagents**	** Cat. No.**	**Source**	

Osteogenesis differentiation medium	RASMX-90021	Cyagen	
Chondrogenesis differentiation medium	RASMX-90041	Cyagen	
Adipogenesis differentiation medium	RASMX-90031	Cyagen	
Cycloheximide (CHX)	2112S	CST	
Wortmannin	9951	CST	
YC-1	ab120915	Abcam	
Thymosin β4	Kindly provided by RegeneRx Biopharmaceuticals		
	Inc. Rockville, MD, United States.		
FG-4497	Synthesized at Fibro Gen,		
	Inc. (San Francisco, CA United States)		
Forward oligonucleotide sequences of TMSB4 primers (Tmsb4x-F)	TGCCGCCGCGATCGCATGTCTGACAAACCCG		
Reverse oligonucleotide sequences of TMSB4 primers (Tmsb4x-R)	CGGCCGCGTACGCGTTTACGATTCGCCAGC		

### Statistical Analysis

Data are expressed as mean ± SE and median. All statistical calculations were performed using the SPSS software (version 14.0; IBM Corporation, Armonk, NY, United States). An independent-sample *t*-test was used to determine differences between the two groups. One-way ANOVA with Dunn’s multiple comparisons test was used to compare the variables between multiple groups. For all analyses, *p* < 0.05 was considered to indicate statistically significant differences.

## Results

### Characterization of Rat BMMSCs

**FIGURE 1:**
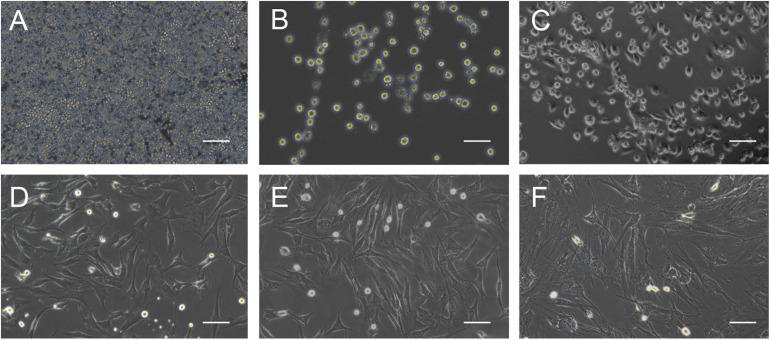
Cell morphology during BMMSCs culture. **(A)** Round mononuclear cells were observed after seeding of 4 h. **(B)** The adherent cells in the shape of short rods were seen 24 h after the first half-volume medium exchange. **(C)** The cells that showed spindle shape and clonal growth were detected after primary culture for 3 days. **(D)** The typical fusiform cells cloned in a fish-like manner were observed on the 7th day of primary culture. **(E)** The third generation of the cultured cells. **(F)** Most of the cells become wide and flat, and granular substances were detected in the cytoplasm after passing through seven times. Scale bar = 200 μm.

The morphology of the cultured BMMSCs was measured by optical microscopy from the beginning of seeding to the seventh passage (Figures 1A–F). The BMMSCs from the SD rats were firmly attached (Figure 1A), and the typical spindle shape was observed 24 h after seeding (Figure 1B). Radial colony tendency (Figure 1C) was shown with continued culture. Fish-like distribution (Figure 1D) was observed when the cells expanded between 70 and 80%. Cells grew vigorously and rapidly at the third passage (Figure 1E) and could be passaged and stabilized over seven passages. After that, the morphology of BMMSCs changed to a flat and enlarged shape (Figure 1F).

The growth curve (Supplementary Figure 2) showed that BMMSCs strictly followed the S growth model, while cells in passages 3–5 expanded faster than the rest. BMMSCs in the third passage were negative for CD34 (Supplementary Figure 3A), CD11B (Supplementary Figure 3B), and CD45 (Supplementary Figure 3C), but positive for CD90 (Supplementary Figure 3D) and CD29 (Supplementary Figure 3E), which was detected by flow cytometry. Furthermore, the cells were positive for CD105 and CD73, and negative for HLA-DR and CD19 (Supplementary Figures 3F–I).

### Tβ4 Promoted Phosphorylation of HIF-1α and Inhibited Degradation

**FIGURE 2:**
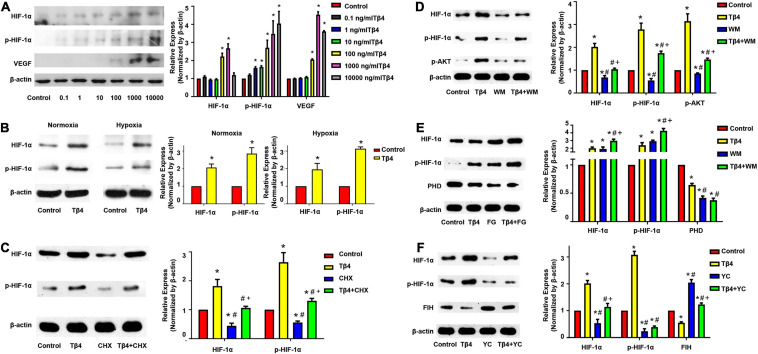
Expression profiling of hypoxic regulatory proteins in BMMSCs. Western blot analyses showing the expression of HIF-1α, phosphorylated HIF-1α (p-HIF-1α), and VEGF in BMMSCs treated with different concentration of Tβ4 **(A)**, in normoxic and hypoxic conditions **(B)**, or with 100 μmol/L cycloheximide (CHX) and 1,000 ng/ml Tβ4 **(C)**. Western blot analyses revealed the expression of the HIF-1, p-HIF-1α, p-AKT **(D)**, PHD **(E)**, and FIH **(F)** in BMMSCs treated with 1,000 ng/ml Tβ4 and/or 0.5 μM wortmannin (WM) **(D)**, 1,000 ng/ml Tβ4 and/or 150 μM FG-4497 (FG) **(E)**, and 1,000 ng/ml Tβ4 and/or 100 μM YC-1 (YC) **(F)**, respectively. The housekeeping protein beta-actin (β-actin) was used for Western blot normalization. The values were presented as means ± SE. Three independent experiments (*n* = 3). One-way ANOVA with Dunn’s multiple comparisons test. *p < 0.05 vs. control for **(A)**; *p < 0.05 vs. red bar; ^#^*p* < 0.05 vs. yellow bar; ^+^*p* < 0.05 vs. blue bar for **(B–F)**. Scale bar = 20 μm for **(B)**.

The BMMSCs were treated with various concentrations of Tβ4 (0.1, 1, 10, 100, 1,000, or 10,000 ng/ml) under normoxia (21% O_2_) for 24 h and then the total cellular protein was isolated and subjected to Western blot analyses. The semi-quantitative Western blot analyses showed that the expression levels of both HIF-1α, phosphorylated HIF-1α (p-HIF-1α), and VEGF were significantly upregulated when the dose increased (Figure 2A).

The BMMSCs were treated with 1,000 ng/ml Tβ4 under normoxia or hypoxia (1% O_2_, 5% CO_2_) for 24 h. Western blot analyses revealed that the expression levels of HIF-1α and p-HIF-1α were significantly upregulated in both normoxic and hypoxic conditions (Figure 2B). The results suggest that Tβ4 promoted HIF-1α and p-HIF-1α protein expressions in an oxygen-independent manner.

To confirm the effect of Tβ4 on HIF-1α protein synthesis, we performed Western blotting after treatment of the BMMSCs with cyclohexamide (CHX), an inhibitor of protein synthesis. The expression of both HIF-1α and p-HIF-1α was decreased after treatment with CHX for 24 h. However, when treated with Tβ4 after 2 h of CHX pretreatment, the level of HIF-1α and p-HIF-1α was not decreased but rather slightly increased (Figure 2C). A previous report showed that BMMSCs protect the myocardium from I/R injury through the PI3K pathway (Angoulvant et al., 2011).

As shown in Figure 2D, AKT phosphorylation was increased when incubated with Tβ4, while the increases of HIF-1α and p-HIF-1α were partly blocked by wortmannin (WM, an inhibitor of PI3K) (Figure 2D). HIF-1α was normoxic degraded by prolyl hydroxylase domain proteins (PHD), and factor-inhibiting HIF (FIH) mediated proteasome system (Brahimi-Horn and Pouyssegur, 2009). We treated the BMMSCs with FG-4497 (a specific PHD inhibitor) and YC1 [3-(5′-hydroxymethyl-2′-furyl)-1-benzyl indazole], an activator of HIF-1α degradation *via* the stimulation of FIH as shown in Figures 2E,F. The expression of PHD was decreased upon Tβ4 treatment and further decreased with the combined treatment of Tβ4 and FG-4497. HIF-1α and p-HIF-1α were significantly increased in the treatment of Tβ4, FG-4497, and Tβ4 + FG compared to the control. It was notably prominent in the treatment of the Tβ4 + FG group (Figure 2E). Similarly, the expression of FIH was decreased with the treatment of Tβ4. In addition, the enhancement of HIF-1α and p-HIF-1α was significantly reduced only in the YC1-treated group and not the Tβ4 + YC1 group (Figure 2F). These results indicate that the increase in HIF-1α and p-HIF-1α was because of increased protein synthesis, reduction of degradation, and partly through the PI3K-AKT pathway.

### TMSB4^*OE*^-BMMSCs Enhance Cardiac Function of the MI Rat

**FIGURE 3:**
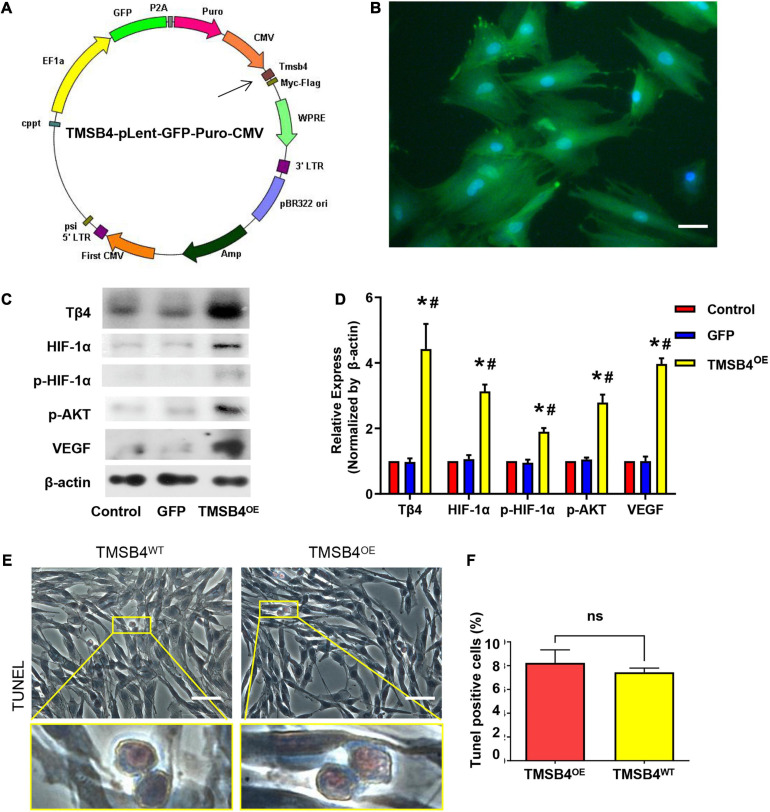
Generation of TMSB4-overexpressing BMMSCs. **(A)** Diagram of the lenti-GFP-Puro-CMV plasmid with the insertion of TMSB4 gene (arrow). **(B)** Immunostaining and fluorescence microscopic analyses of TMSB4^*OE*^-BMMSCs displayed the expression pattern of GFP (green). The cells were also stained for nuclei (blue, DAPI). Scale bar = 20 μm. **(C)** The total cellular protein was then extracted from the BMMSCs with or without TMSB4 overexpression and subjected to Western blot analyses, which revealed the expression pattern of Tβ4, HIF-1α, p-HIF-1α, p-AKT, and VEGF protein. **(D)** Semi-quantitative Western blot analyses demonstrated that the expression level of Tβ4, HIF-1α, p-HIF-1α, p-AKT, and VEGF were significantly upregulated in the case of TMSB4^*OE*^-BMMSCs than TMSB4^*WT*^-BMMSCs (control) group and GFP-overexpressing BMMSCs (three independent experiments, one-way ANOVA with Dunn’s multiple comparisons test; *p < 0.05 vs. control; ^#^*p* < 0.05 vs. GFP). Immunostaining of TMSB4^*WT*^-BMMSCs **(E)** and TMSB4^*OE*^-BMMSCs **(F)** displayed the expression pattern of TUNEL. Scale bar = 50 μm.

**FIGURE 4:**
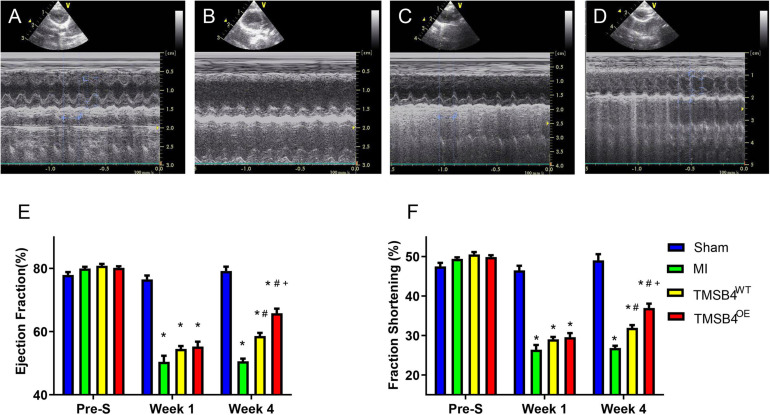
Assessment of cardiac function. Sham-operated control rats **(A)**, MI-only control rats **(B)**, and those treated with intramyocardial injections of TMSB4^*WT*^-BMMSCs **(C)** and TMSB4^*OE*^-BMMSCs **(D)** were subjected to echocardiographic assessments of left ventricular (LV) function on day 28 following the different treatments. Ejection fraction (EF) **(E)** and fractional shortening (FS) **(F)** were assessed before MI induction (pre-S) and on post-treatment weeks 2 and 4. Data are given as means ± SE. Nine animals per group (one-way ANOVA with Dunn’s multiple comparisons test; *p < 0.01 vs. sham; ^#^*p* < 0.01 vs. MI; ^+^*p* < 0.05 vs. TMSB4^*WT*^-BMMSCs).

TMSB4-overexpressing (TMSB4^*OE*^) BMMSCs were successfully established (Figures 3A–D). The expression of Tβ4, HIF-1α, p-HIF-1α, p-AKT, and VEGF were significantly upregulated in TMSB4^*OE*^ cells (Figures 3C,D). To explore if the high-level intracellular HIF-1a would lead to cytotoxicity, TUNEL staining of TMSB4^*OE*^-BMMSCs and TMSB4^*WT*^-BMMSCs was carried out, which showed no significant intergroup differences (Figures 3E,F). The biological identification of BMMSC-TMSB4^*OE*^ based on surface markers was carried out along with tripotent differentiation; no significant intergroup differences were detected (Supplementary Figures 3, 4). The left ventricle functional parameters were evaluated in rats intramyocardially injected with TMSB4^*OE*^-BMMSCs or TMSB4^*WT*^-BMMSCs after surgically induced MI to determine the effect of TMSB4-overexpressed BMMSCs on heart function. MI was induced by permanently ligating the anterior descending coronary artery. After 1 week, cells (3 × 10^6^ cells/animal) were intramyocardially injected into three sites (1 × 10^6^ cells/site) in the border zone of the anterior wall of the LV. The third group of animals (the MI group) was treated with equivalent injections of cell-free PBS after MI injury, while the animals in the Sham group underwent all surgical procedures for MI induction except for the ligation step. Echocardiographic measurements (Figures 4A–D) of LVEF (Figure 4E) and FS (Figure 4F) 4 weeks after treatments showed that LV function was significantly greater in both cell-treatment groups than in the MI group. It was also more prominent in the TMSB4^*OE*^-BMMSC-treated than TMSB4^*WT*^-BMMSCs-treated animals. The other parameters detected in echocardiography included the heart rate, end-diastolic diameter, and anterior and posterior wall thickness (Supplementary Figure 5).

### TMSB4^*OE*^-BMMSCs Smaller Infarct Size, Apoptosis, and Hypertrophy After MI Than TMSB4^*WT*^-BMMSCs

**FIGURE 5:**
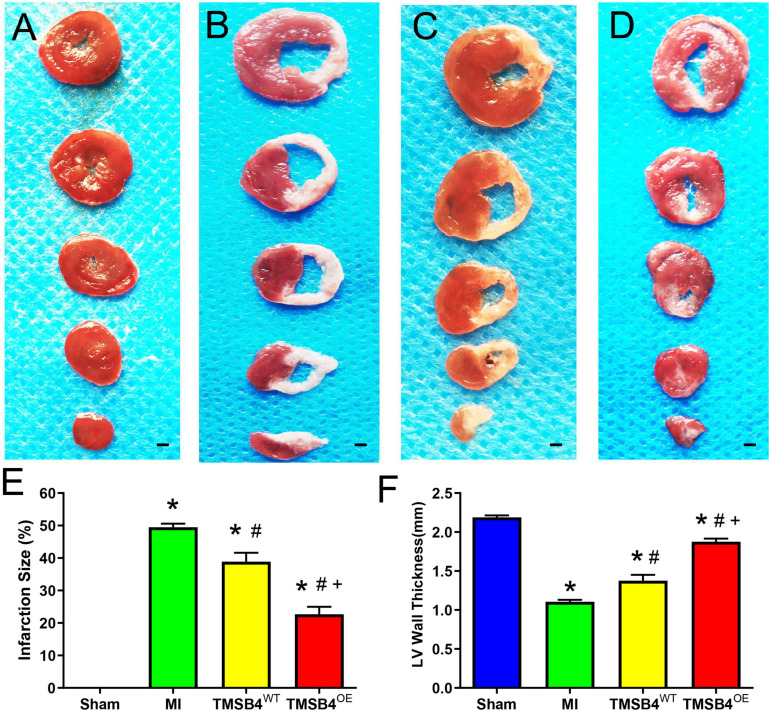
Assessment of infarct size and left ventricular morphology. TTC staining showed the areas of infarcted (white, non-viable) and non-infarcted (red, viable) zones in post-treatment day 28 ventricular tissue sections in Sham-operated control rats **(A)**, MI-only control rats **(B)**, and those treated with TMSB4^*WT*^-BMMSCs **(C)** and TMSB4^*OE*^-BMMSCs **(D)**. The infarct size was quantified as the ratio of the scar area to the total surface area of the left ventricle and expressed as a percentage **(E)** and quantified as left anterior wall thickness **(F)**. Data are presented as means ± SE. Nine animals per group (one-way ANOVA with Dunn’s multiple comparisons test; *p < 0.01 vs. sham; ^#^*p* < 0.05 vs. MI; ^+^*p* < 0.05 vs. TMSB4^*WT*^-BMMSCs. Scale bar = 1 mm for panels **(A–D)**.

**FIGURE 6:**
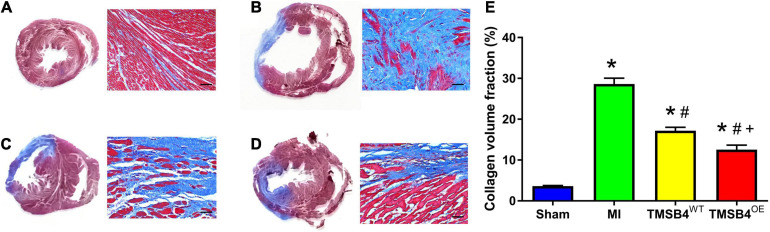
Assessment of collagen volume. Masson staining showed the areas of fibrotic (blue, non-viable) and non-fibrotic (red, viable) zones in post-treatment day 28 ventricular tissue sections in Sham **(A)**, MI **(B)**, TMSB4^*WT*^-BMMSCs **(C)**, and TMSB4^*OE*^-BMMSCs **(D)** treated groups. The collagen volume was quantified as the area occupied by connective tissue divided by the sum of the areas and expressed as a percentage **(E)**. Data are presented as means ± SE. Nine animals per group. One-way ANOVA with Dunn’s multiple comparisons test. *p < 0.01 vs. sham; ^#^*p* < 0.05 vs. MI; ^+^*p* < 0.05 vs. TMSB4^*WT*^-BMMSCs. Scale bar = 100 μm.

The infarct size from each group was assessed by TTC staining (Figures 5A–D) and showed that the TMSB4^*OE*^-BMMSCs-treated animals exhibited more significant reductions in infarct size (Figure 5E) and had greater LV wall thickness (Figure 5F) than TMSB4^*WT*^-BMMSCs-treated and untreated controls subjected to MI. This cardiac recovery effect was corroborated by a significant reduction in collagen volume fraction with Masson staining (Figures 6A–E).

**FIGURE 7:**
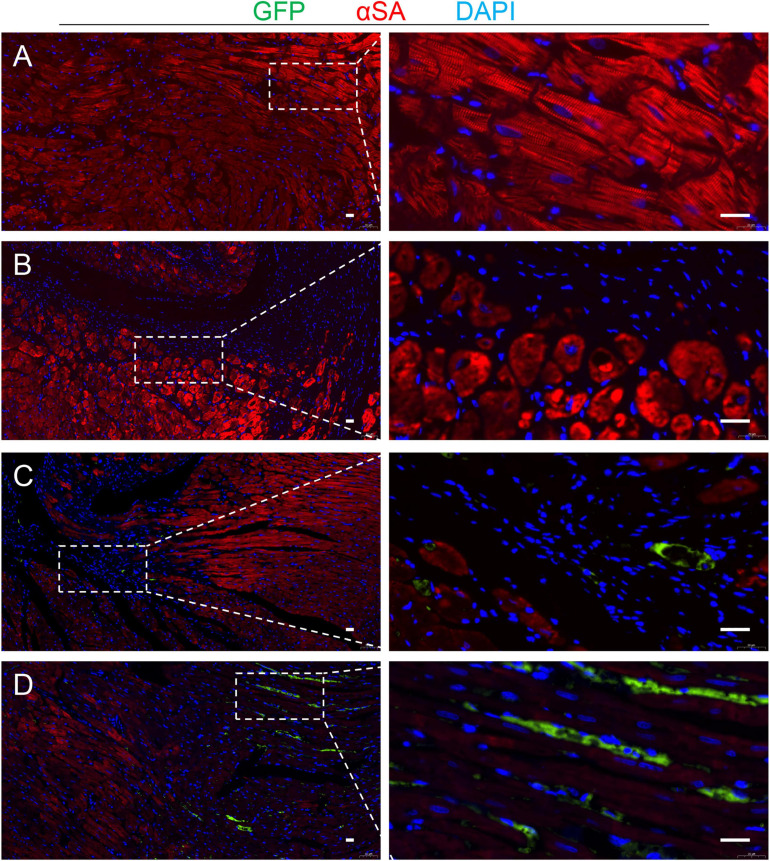
Detection of the transplanted cells. Serial sections from the hearts of TMSB4^*OE*^-BMMSCs-treated rats that were sacrificed 4 weeks after MI induction were stained for the presence of GFP and α-sarcomeric actin (αSA), and the nuclei were counterstained with DAPI in Sham **(A)**, TMSB4^*WT*^-BMMSCs **(B)** and TMSB4^*OE*^-BMMSCs **(C,D)** treated groups. Then, the transplanted cells were defined as the cells that expressed GFP and DAPI. GFP was undetected in the TMSB4^*WT*^-BMMSCs-treated hearts. Five randomly selected viewing fields were evaluated per section, and 20 sections were evaluated per animal. Scale bar = 20 μm.

**FIGURE 8:**
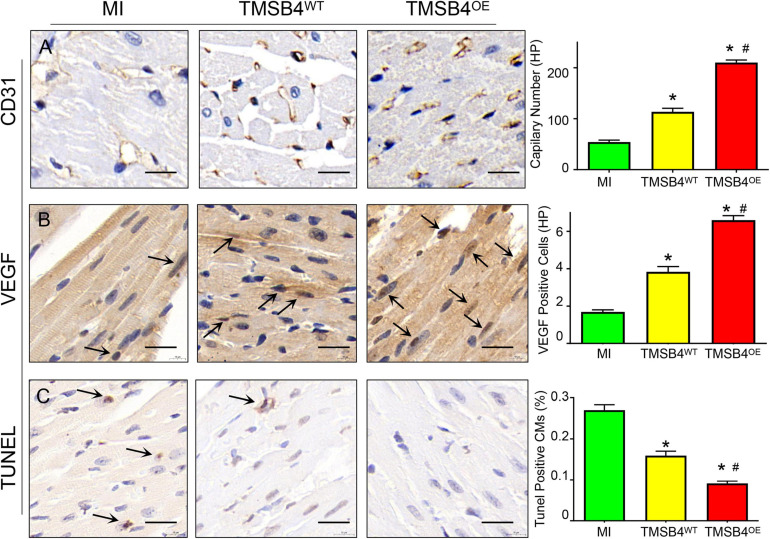
Assessment of angiogenesis and apoptosis. Serial sections from the hearts of MI, TMSB4WT- BMMSCs-, and TMSB4OE-BMMSCs-treated rats that were sacrificed 4 weeks after MI induction were immunohistochemically stained for the presence of CD31 **(A)**, VEGF **(B)**, and the TUNEL assay **(C)**. Capillary number was quantified as the number of CD31-positive vascular structures in the peri-infarcted zone per high-power field [**(A)**, *p < 0.01 vs. MI; ^#^*p* < 0.05 vs. TMSB4WT-BMMSCs]; expression of VEGF was quantified as the number of VEGF-positive cells in the peri-infarcted zone per high-power field [**(B)**, *p < 0.01 vs. MI; ^#^*p* < 0.05 vs. TMSB4WT-BMMSCs]; apoptotic cardiomyocytes were quantified as the number of TUNEL-positive cardiomyocytes divided by the total number of cardiomyocytes and expressed as a percentage [**(C)**, *p < 0.05 vs. MI; ^#^*p* < 0.05 vs. TMSB4WT-BMMSCs]. Data are given as means ± SE. Nine animals per group (one-way ANOVA with Dunn’s multiple comparisons test. Scale bar = 20 μm).

TMSB4^*OE*^-BMMSCs were detected in the heart’s border zone 4 weeks after transplantation (Figure 7). Next, the neo-angiogenic response assessment in MI rats was evaluated by immunostaining, using endothelial phenotypic markers, i.e., CD31 (Figure 8A) and VEGF expression (Figure 8B). The TMSB4^*OE*^-BMMSCs-treated animals showed significantly elevated vessel density and VEGF expression compared to the TMSB4^*WT*^-BMMSCs-treated and untreated MI groups (Figures 8A,B). Finally, the cardiomyocyte apoptosis assessment in MI rats was evaluated by TUNEL immunostaining. The number of TUNEL-positive cardiomyocytes was significantly smaller in both cell-treatment groups than in MI rats, which was most prominent in BMMSC-TMSB4^*OE*^-treated animals (Figure 8C).

## Discussion

In the present study, we found for the first time that BMMSCs transfected with pro-angiogenic gene (*TMSB4*) significantly improved the cardiac function and infarct size in rat post-MI heart (Figures 4, 5). Improvement in the heart function was accompanied by a significant enhancement of angiogenesis. Intracoronary infusion of autologous bone marrow cells (BMCs) has been proposed as a therapeutic strategy to enhance tissue perfusion, reduce scar formation, and improve heart function after MI (Wollert and Drexler, 2010). The identification of paracrine-acting proteins, including well-known cytokines, chemokines, and growth factors, acts as a central mechanism where cell-based therapies improve tissue perfusion and contractile functions (Jay and Lee, 2013). Angiogenesis or neovascularization, the first step of tissue repair, plays a critical role in promoting myocardial regeneration in patients with cardiac disease (Mathison and Rosengart, 2018). Inducing angiogenesis is a novel approach for the functional recovery of ischemic tissues (Bonauer et al., 2009; Korf-Klingebiel et al., 2015). Tβ4 is a potent stimulator of coronary vasculogenesis and angiogenesis. Thus, pre-treating hearts using Tβ4 might further improve cardiac function and scar area (Smart et al., 2007a; Qian et al., 2012). In the process of ischemic heart disease treatment, the strategy of using donor cells with target genes to structurally rebuild the ventricular wall likely has favorable prospects in forthcoming biotherapies (Tang et al., 2013).

We have previously shown that transplantation of VEGF or SHH gene-transfected MSCs can better improve myocardial perfusion and restore heart function than either cellular or gene therapy alone (Yang et al., 2007, 2009). Therapeutic neovascularization can be achieved in the adult organism through either protein application, gene overexpression, or cell therapy. Preclinical and clinical data suggest that therapeutic neovascularization is achievable but requires novel factors that induce both capillary growth and vessel maturation to induce functional neovascularization. Thymosin β4 (Tβ4) improves wound healing *via* a variety of different mechanisms, namely, enhanced angiogenesis, improved keratinocyte migration, collagen deposition, as well as wound contracture. In addition, Tβ4 has anti-inflammatory properties. Thymosin β4 is essential not only for vascular development but also for cardiomyocyte differentiation and maturation. The combination of Tβ4 and adeno-associated viruses (AAV) was tested in translational large animals of chronic myocardial ischemia with or without cardiovascular risk factors. Thymosin β4 could induce therapeutic neovascularization in wild-type pigs as well as in pigs suffering from diabetes mellitus (Hinkel et al., 2018). To study whether prolonged release is necessary for observed cardioprotection, we tried intramyocardial injection of Tβ4 peptide (400 μg in 150 μl PBS) immediately after the LAD ligation procedure. All animals were assessed 4 weeks after treatment. Interestingly, we did not observe a similar cardioprotection following the direct intramyocardial injection of Tβ4 (data not shown). It is reasonable to believe that the cardioprotective effect could hardly be achieved by a single shot of the peptide, which would likely be squeezed and rapidly washed away. This finding was in accordance with the report by Bock-Marquette et al., which suggested that a prolonged release of these chemicals is important for the chemicals to exert their cardioprotective effects. In the present study, we found that TMSB4^*OE*^-BMMSCs enhance angiogenesis and reduce the cardiac infarct size, which results in a significant induction of cardiac recovery in the post-MI rat. Notably, lower collagen deposits were partly because of angiogenesis and possibly reduced inflammation and oxidative stress. The defects in the molecular pathways responsible for suppression and resolution of the post-infarction inflammatory reaction may be involved in the pathogenesis of adverse remodeling and heart failure following MI. *In vitro* studies have suggested that TGF-β1-induced myofibroblast conversion may be mediated through both canonical Smad-dependent and Smad-independent signaling pathways. Moreover, neutralization experiments using gene therapy with the extracellular domain of the type II TGF-β receptor in a model of MI suggested that early inhibition may worsen dysfunction, accentuating the inflammatory response, while late disruption of TGF-β signaling may protect from interstitial fibrosis and hypertrophic remodeling (Prabhu and Frangogiannis, 2016). Over the past 15 years, several studies have contributed toward our understanding of the mechanism of Tβ4 function; it is now recognized that Tβ4 is involved in a wide range of cellular processes aside from regulating cytoskeletal assembly. The most notable of genes from an angiogenic perspective is probably *VEGF*. An upregulation of *VEGF* was first described following overexpression of Tβ4 in B16-F10 lung tumor cells; conversely, a downregulation of *VEGF in situ* was observed in Tβ4 knockdown hearts, suggesting that appropriate *VEGF* expression may require *T*β*4* (Smart et al., 2007b). In the present study, VEGF was detected significantly enhanced by exogenous Tβ4 (Figure 2A) as well as the Tβ4-overexpressed BMMSCs (Figure 3C). Furthermore, we found that *T*β*4* induces angiogenesis by stabilizing HIF-1α protein in an oxygen-independent manner, which is consistent with existing literature (Jo et al., 2010; Ock et al., 2012).

HIF-1α was normoxic degraded by prolyl hydroxylase domain proteins (PHD), and FIH mediated proteasome system. Thus, we detected the degradation effects of Tβ4 treatment and found that enhancement of HIF-1α and p-HIF-1α proteins was blocked by FIH promoter (YC-1). Moreover, the expression of PHD was decreased with the treatment of Tβ4 and further decreased when combined with Tβ4 and FG-4497 (Figure 2). These results show that the increase in HIF-1α and p-HIF-1α was due to increased protein synthesis and reduced degradation and partly through the PI3K-AKT pathway.

Our study has some limitations. Apart from the anti-fibrotic and pro-angiogenetic potential, intramyocardial transplantation of MSCs improves cardiac repair by promoting the polarization of macrophages and increasing the induction of Tregs, thereby regulating immune response as well. In this study, we did not detect the expression of HIF-1α and p-HIF-1α *in vivo* and the other cardioprotective effects of TMSB4^*OE*^-BMMSCs such as inflammation, immunity, myocardial hypertrophy, cell migration, and proliferation. Future studies should consider investigating the therapeutic role of the *T*β*4* gene in the PI3K-AKT pathway.

## Conclusion

Our data suggest that 4 weeks after MI treatment, significant repair of an injured LV can be achieved by a novel BMMSC line showing TMSB4 overexpression. The small infarct size observed in TMSB4^*OE*^-BMMSCs-treated animals can also lead to a corresponding increase in the activation of paracrine mechanisms such as the increase in angiogenesis at the border zone, 4 weeks after transplantation. This increase in paracrine activity may also contribute to improvements in LV remodeling and LV chamber function. Furthermore, the increase in HIF-1α and p-HIF-1α induced by *T*β*4* was partly because of an increase in protein synthesis *via* the AKT pathway and the reduction of degradation *via* the PHD and FIH pathways, which may serve as potential therapeutic targets for the treatment of MI.

## Data Availability Statement

The raw data supporting the conclusions of this article will be made available by the authors, without undue reservation.

## Ethics Statement

The animal study was reviewed and approved by the Research Ethics Committee of Second Xiangya Hospital.

## Author Contributions

ST carried out data collection and/or assembly of data, data analysis, and wrote the manuscript. ST, WZ, ZZ, MW, WC, and XW carried out data collection. ZL, JP, and JY carried out data analysis and interpretation and manuscript revision. CF and JY designed and conceptualized the study and carried out manuscript revision. All authors read and approved the final manuscript.

## Conflict of Interest

The authors declare that the research was conducted in the absence of any commercial or financial relationships that could be construed as a potential conflict of interest.

## Abbreviations

T β 4: thymosin beta-4
HIF: hypoxia-inducible factor
p-HIF -1 α: phosphorylated HIF-1 α
FIH: factor-inhibiting HIF
CHX: cycloheximide
WM: wortmannin
PHD: prolyl hydroxylase domain proteins
YC1: 3-(5 ′ -hydroxymethyl-2 ′ -furyl)-1-benzyl indazole
BMMSC: bone marrow mesenchymal stromal cell
BMMSC-TMSB4^*O**E*^: TMSB4-overexpressed BMMSCs
BMMSC-TMSB4^*W**T*^: wild-type BMMSCs which express normal levels of TMSB4
MI: myocardial infarction
TTC: 2,3,5-triphenyltetrazolium chloride
LAD: left anterior descending branch of coronary artery
β-actin: beta-actin
EF: ejection fraction (left ventricular)
FS: fractional shortening.
