# Supplementary material for: TMSB4 Overexpression Enhances the Potency of Marrow Mesenchymal Stromal Cells for Myocardial Repair
Source: Front Cell Dev Biol. 2021 Jun 9;9:670913. doi: 10.3389/fcell.2021.670913 (PMC8221609; doi:10.3389/fcell.2021.670913)
Supplement: Supplementary file 1 [file Data_Sheet_1.doc]

TMSB4 Overexpression Enhances the Potency of Marrow Mesenchymal Stromal Cells for Myocardial Repair

**Supplemental figure legends**

**Supplemental figure 1: Heart tissue management.**

The explanted hearts were collected on the 28^th^-day post-treatment. In order to massimize the usage, The fresh heart was cut into five slices from the ligation size to the apex and each slice was cut off a thinner slice which used for TTC staining and the rests were used for IHC and IF.

**Supplemental figure 2: Growth patterns of BMMSCs in various generations.**

All generations of BMMSCs showed a representative S-shape proliferation pattern (retention period, logarithmic growth period, and plateau period).

**Supplemental figure 3:** The cellular markers from the BMMSC-TMSB4^OE^ were detected via flow cytometry for the expression of CD34 (A), CD11B (B), CD45 (C), CD90 (D), CD29 (E), CD105 (F), CD73 (G), HLA-DR (H) and CD19 (I), and the possitive rate for CD34, CD11B, CD45, CD90, CD29, CD105, CD73, HLA-DR and CD19 were 0%, 12.4%, 0.6%, 99.8%, 99.0%, 74.2%, 25.03%, 0.70% and 0.96%, respectively. Isotype was used as control.

**Supplemental figure 4:** The tripotent differentiation of osteogenesis, chondrogenesis and adipogenesis from the BMMSC-TMSB4^OE^ were assessed via mineralized nodes with alizarin red staining (red), mucopolysaccharides with alcian blue staining (blue) and lipids with oil red O staining (red), respectively.

**Supplemental figure 5:** On day 28 following the different treatments, the other parameters in assessment of cardiac function including the heart rate, left ventricular end diastolic anterior wall thicknes (LVAWd), left ventricular end systolic anterior wall thicknes (LVAWs), left ventricular internal diameter at end-diastole (LVIDd), diastolic thickness of left ventricular posterior wall (LVPWd), systolic thickness of left ventricular posterior wall (LVPWs), end-diastolic volume (EDV) and end-systolic volume (ESV).
